# Clinicopathological Significance of Defective DNA Mismatch Repair in Endometrial Carcinoma: A Single-Center Study From Bahrain

**DOI:** 10.7759/cureus.67332

**Published:** 2024-08-20

**Authors:** Maryam Hammad, Sayed Ali I Almahari, Shri Umakanth, Zainab A Toorani

**Affiliations:** 1 Pathology, Salmaniya Medical Complex, Manama, BHR; 2 Pathology and Laboratory Medicine, Salmaniya Medical Complex, Manama, BHR; 3 Pathology, RCSI Bahrain, Manama, BHR; 4 Pathology, Salmaniya Medical Complex, Saar, BHR

**Keywords:** endometrioid carcinoma, lynch syndrom, serous carcinoma, mmr deficient, endometrial carcinoma

## Abstract

Introduction: Endometrial carcinoma, the most prevalent gynecologic malignancy in developed countries, represents a significant public health issue worldwide. DNA mismatch repair (dMMR) deficiency is an important molecular mechanism in endometrial carcinoma development, clinical course, and prognosis.

Aims and objectives: This study aimed to determine the incidence and histological subtypes of endometrial carcinoma among Bahraini women, evaluate the prevalence of MMR deficiency using immunohistochemistry in these patients and analyze the association between MMR deficiency and clinicopathological features, including potential links to Lynch syndrome.

Patients and methods: This single-center retrospective study included 115 endometrial carcinoma patients diagnosed between January 2020 to June 2023. Immunohistochemistry was used to assess the expression of the four main MMR proteins (MLH1, MSH2, MSH6, PMS2). Clinicopathological features and survival outcomes were compared between MMR-deficient and MMR-proficient tumors. Medical records of patients were retrieved from I-SEHA system. Statistical analysis was done using SPSS.

Results: The study included a wide age range of patients, with a mean age of 59.5 years. The majority were Bahraini nationals. Endometrioid carcinoma was the most common histologic subtype (73%), followed by serous carcinoma (8.7%). Most patients presented with early-stage disease (76.8% stage I), and 39.8% had low-grade tumors. Significant proportions of cases showed loss of expression of mismatch repair (MMR) proteins MLH1 (24.2%), PMS2 (25%), MSH6 (14.5%), and MSH2 (12.7%), without significant associations with age.

Conclusion: This study found endometrial cancer to be a significant health concern in Bahrain, with a relatively high prevalence and younger age of onset compared to global averages. The data shows a predominance of endometrioid subtype and higher-grade tumors. Notably, a substantial proportion exhibited MMR deficiency, an important biomarker. These findings suggest the need for enhanced screening, early detection, and tailored treatment approaches in Bahrain. Further research and robust national cancer registries are warranted to fully understand the underlying risk factors and guide evidence-based interventions to mitigate the burden of this disease.

## Introduction

Endometrial carcinoma is the most prevalent gynecologic malignancy in developed countries, representing over 75% of all gynecologic cancers and a significant public health issue worldwide [1]. Over recent years, a vast amount of evidence has shown a central role of deficient DNA mismatch repair (dMMR) in the mutagenesis of several malignancies, including endometrial carcinoma. Studies have found that up to 25-30% of endometrial cancers exhibit dMMR, which leads to a hypermutated phenotype and is associated with the development of the type I, estrogen-related subtype of endometrial cancer [2]. dMMR corrects errors such as base-base mismatches, insertion/deletion loops, and slippage errors that occur during DNA replication, preserving genomic integrity. However, the inactivation of this system as a result of mutations or epigenetic modifications in the dMMR genes, such as MLH1, MSH2, MSH6, and PMS2, results in the hazardous accumulation of errors, genomic destabilization, and can prompt tumorigenesis [3]. While the essence of dMMR in colorectal carcinoma is very well studied, its clinical relevance and importance in endometrial carcinoma have not been explored thoroughly and remain vague, especially in Bahrain and the Middle Eastern region [4].

The Cancer Genome Atlas (TCGA) has established a novel molecular classification to further subcategorize the subtypes of endometrial cancer and also to predict prognosis [5,6]. This classification divides endometrial carcinomas into four groups based on immunohistochemical (IHC) stains and molecular studies: (1) mismatch repair (MMR)-deficient: Characterized by deficient dMMR proteins; (2) p53-abnormal: Exhibits aberrant p53 expression; (3) p16-abnormal: Shows abnormal p16 expression; (4) MMR-proficient/p53-normal/p16-normal: No significant molecular alterations in these markers.

This single-center study in Bahrain aims to investigate the clinicopathological significance of dMMR in endometrial carcinoma patients as it is one of the most prevalent gynecological malignancies worldwide, and understanding the molecular subtypes can help guide treatment and management strategies [6]. There are many studies regarding endometrial cancer worldwide, however, there is a paucity of published data from Bahrain. Hence, this study was conducted to evaluate the clinicopathological parameters of endometrial carcinoma in the Bahraini population.

## Materials and methods

Study design and setting

This was a retrospective, observational study conducted at the Salmaniya Medical Complex, the main tertiary care center in Bahrain. The study period spanned from January 2020 to June 2023.

Study population and data collection

All cases of endometrial carcinoma diagnosed during the study period were identified and retrieved from the I-Seha electronic medical record system. The I-Seha system contains comprehensive histopathology reports for the Bahraini population, providing detailed information on the diagnosis and management of endometrial cancer cases.

The inclusion criteria for this study were patients diagnosed with endometrial carcinoma during the study period, and availability of complete clinicopathological and IHC data. Cases were excluded if histological subtype was other than endometrial carcinoma (e.g. sarcoma), or if insufficient clinical or pathological information was available in the medical records.

Histopathological assessment

The specific histological types of endometrial cancer were identified and classified according to the World Health Organization (WHO) criteria. IHC analysis was performed on formalin-fixed, paraffin-embedded tumor samples using antibodies against the four MMR proteins: MLH1, MSH2, MSH6, and PMS2. Tumors were considered MMR deficient if there was a complete loss of expression of any of the four MMR proteins on immunohistochemistry.

Clinicopathological data collection

Detailed clinicopathological information was collected from the medical records, including patient age, histological subtype, tumor grade, International Federation of Gynecology and Obstetrics (FIGO) stage, and treatment modalities.

Statistical analysis

Statistical analysis was performed using the SPSS software system. Relationships between MMR status and clinicopathological variables were investigated using appropriate statistical tests, such as chi-square or Fisher's exact tests for categorical variables and t-tests or Mann-Whitney U tests for continuous variables. The impact of MMR status on patient outcomes, including overall survival, was evaluated using Kaplan-Meier survival analysis and log-rank tests. A p-value of less than 0.05 was considered statistically significant.

Ethical considerations

The study protocol was approved by the Institutional Review Board of the Government Hospitals - Bahrain. 

## Results

Bahrain has a lower prevalence of endometrial cancer compared to global averages. At 6.77 cases per 100,000 population, it falls below the worldwide incidence of 14.7 per 100,000 women reported in literature [7,8]. This difference could be due to various factors, including lifestyle habits, hormonal factors, and even access to healthcare screening.

The current data might not provide a complete picture. While this single-center study provides valuable insights into the clinicopathological significance of dMMR in endometrial carcinoma patients in Bahrain, nationwide epidemiological data would be needed to fully understand the disease burden and distribution of molecular subtypes across the country. Factors such as patient demographics, geographic variations, and access to healthcare services could all impact the prevalence and characteristics of endometrial cancer in Bahrain and should be investigated in future large-scale studies.

Patients’ characteristics

The study included patients aged between 29 and 82 years, a wide range covering both younger and older individuals. The average age of the patients was 59.50 years, indicating that the majority were in the middle to older age groups. Younger individuals (≤40 years) made up a small portion (7.8%), indicating lower prevalence in this age group. Patients under 50 years also formed a relatively small percentage (15.6%), further suggesting that the disease is more prevalent in older age groups. Interestingly, a significant number (45.2%) were under 60, highlighting a presence in younger to middle-aged groups. The largest group (under 80 years) accounted for almost 98%, with the majority being younger than 80.

The relatively small proportion of patients under 40 years old indicates that endometrial cancer is less prevalent in the younger population. This single-center retrospective study included 115 endometrial carcinoma patients diagnosed between January 2020 to June 2023. The majority (91.3%) were Bahraini nationals, reflecting the country's demographics. 

Histologic subtypes and clinical presentation

Endometrial biopsy was the most common diagnostic method in all the cases. About (45.6%) of them were not followed up by surgical excision and surgical resection was done in the remaining patients (54.4%).

Regarding histologic types of cancers (Table 1). The majority (73%) were endometrioid carcinomas, the most common type globally [7,8]. Serous carcinomas were the next most common (8.7%), while other less frequent types (18.3%) included clear cell carcinomas, carcinosarcomas, and rare high-grade undifferentiated carcinomas.

**Table 1:** Histologic types of endometrial cancers

Type	Frequency (Percentage)
Endometrioid Carcinoma	84 (73%)
Serous Carcinoma	10 (8.7%)
Others	21 (18.3%)

Abnormal uterine bleeding was the most frequent symptom (60%), emphasizing its importance as a warning sign. Other symptoms included a uterine mass, abnormal radiological findings, and malignant fluid buildup in the abdomen (peritoneal effusions) in advanced cases.

Grade and stage

As shown in the Table 2, the majority of patients (76.8%) had early-stage disease (FIGO stage 1). A smaller proportion (14.3%) presented with stage 2 disease, and a small number (8.9%) had advanced localized disease.

In terms of tumor grade, 39.8% of patients had low-grade tumors, 30.7% had high-grade tumors, and the remaining 29.5% had intermediate-grade tumors.

**Table 2:** Stage and grade of endometrial cancers FIGO:  International Federation of Gynecology and Obstetrics

FIGO Stage	Percentage
Stage 1	76.80%
Stage 2	14.30%
Stage 3	8.90%
FIGO grade	Percentage
Grade 1	39.80%
Grade 2	30.70%
Grade 3	29.50%

MMR protein status

A key finding of the present study was the detailed analysis of MMR protein expression in endometrial carcinoma cases. The MMR system is responsible for correcting DNA replication errors, and its deficiency is associated with specific molecular and clinicopathological characteristics of endometrial cancers.

**Figure 1:**
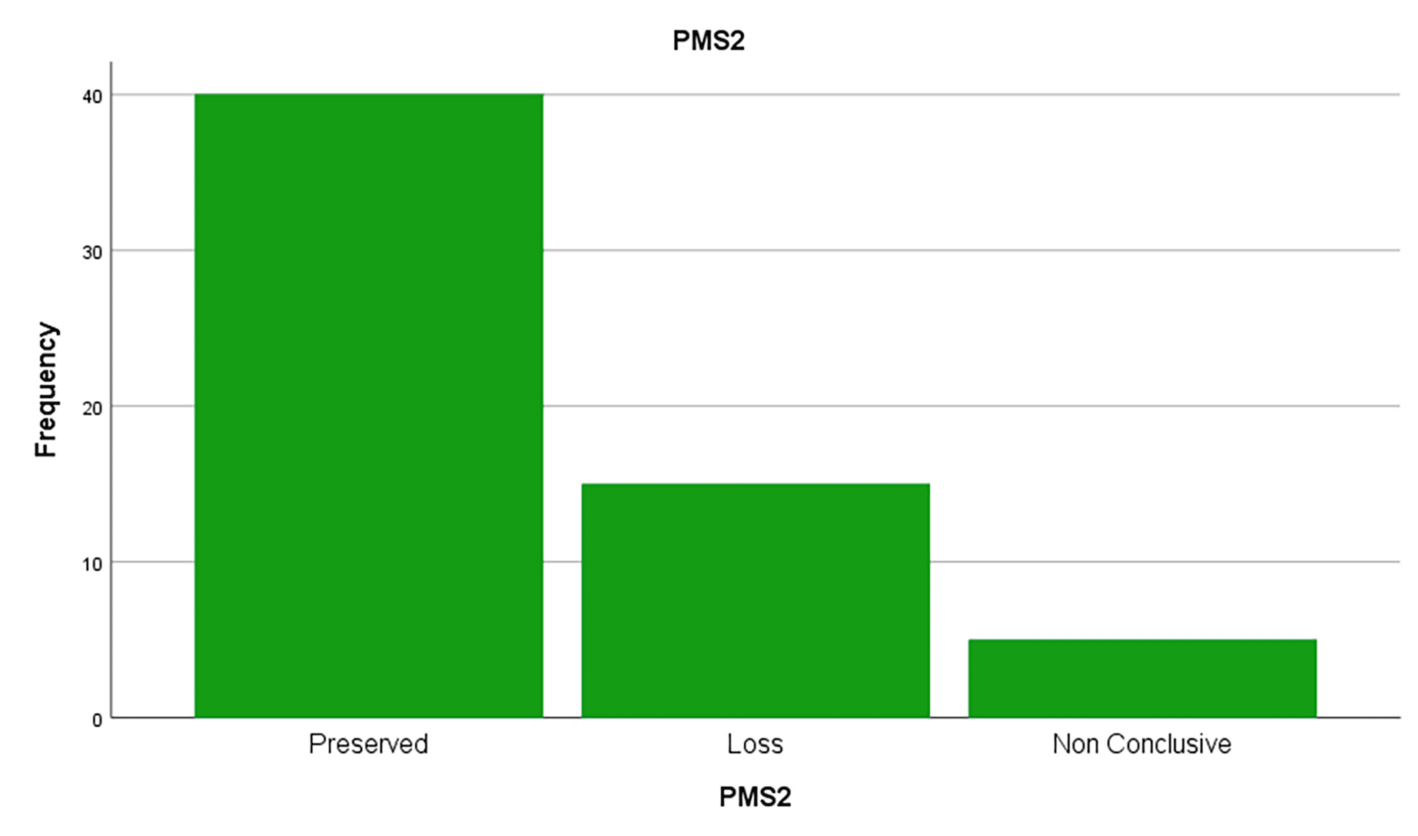
PMS2 protein loss frequencies in the sample studied

**Figure 2:**
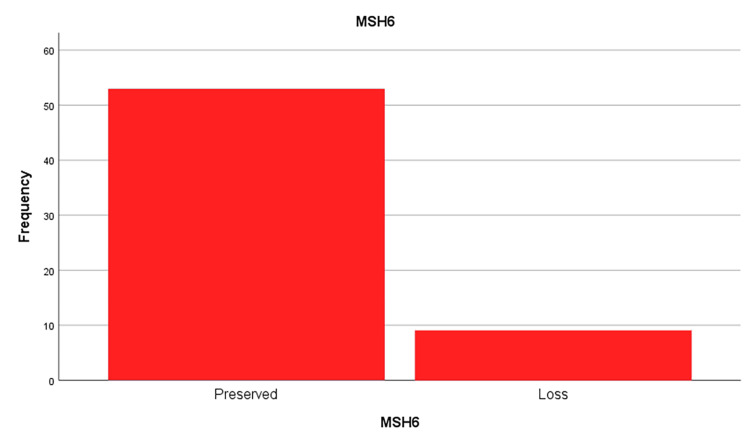
MSH6 protein loss frequencies in the sample studied

**Figure 3:**
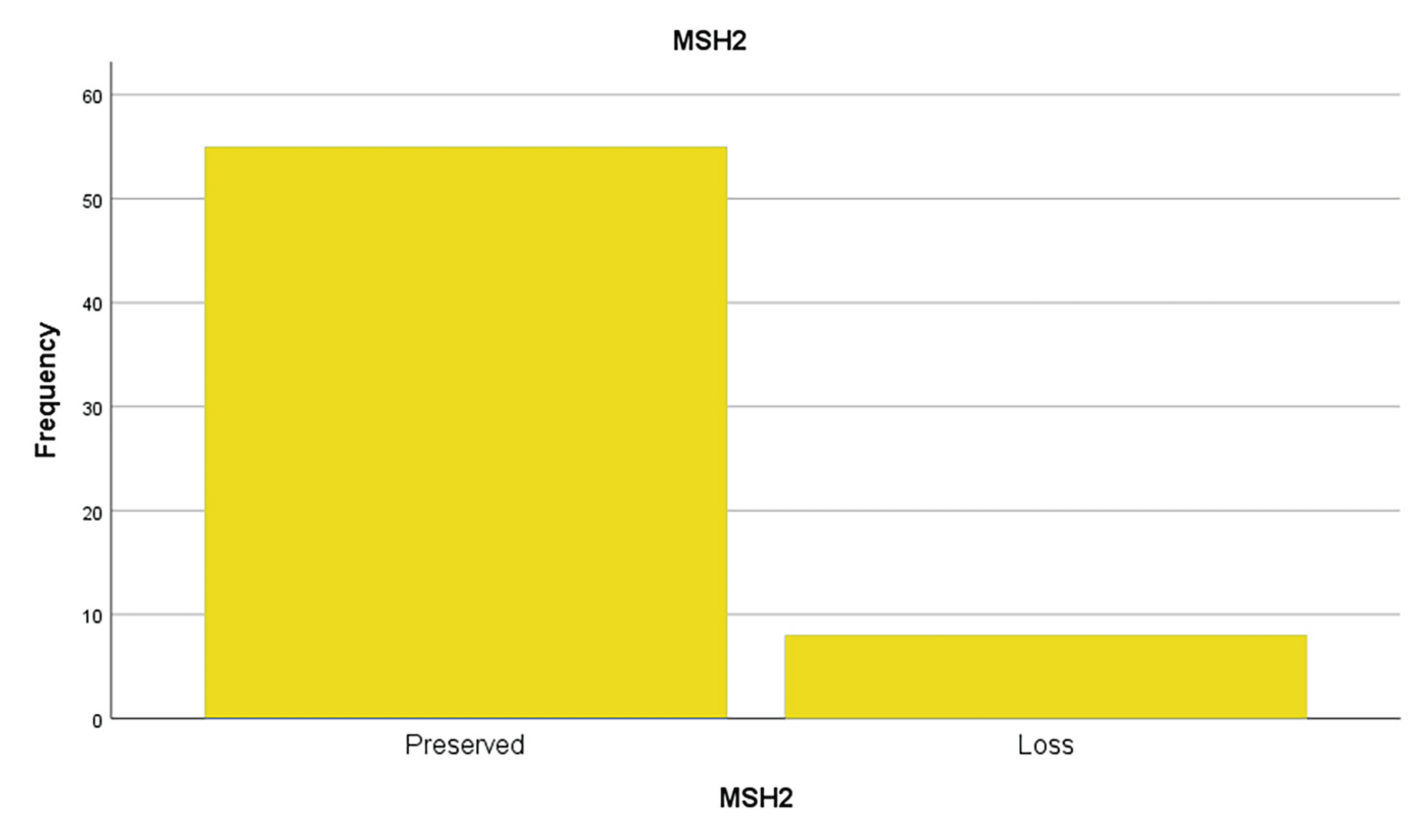
MSH2 protein loss frequencies in the sample studied

**Figure 4:**
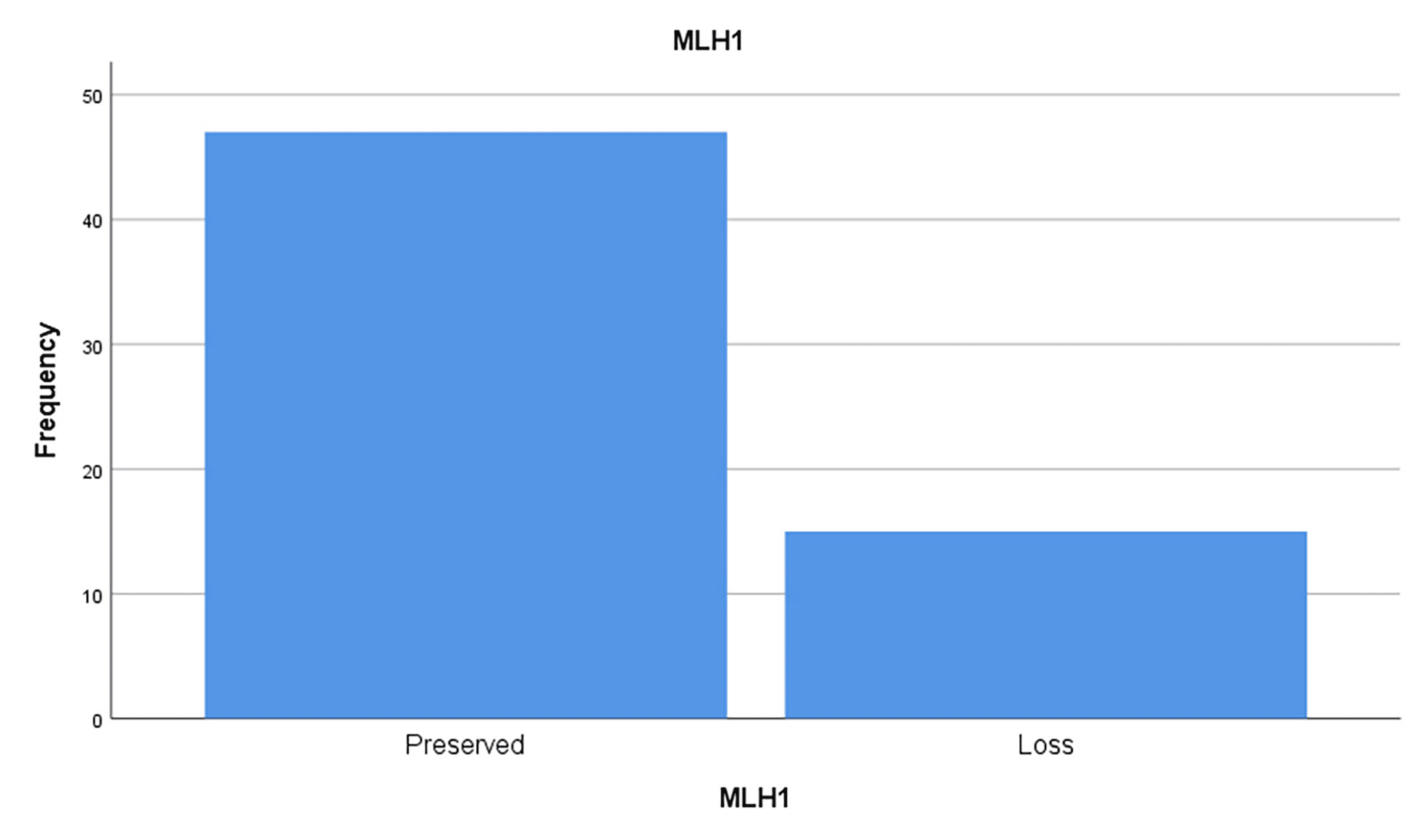
MLH1 protein loss frequencies in the sample studied

This study evaluated the MMR protein status for four key MMR proteins: MLH1, PMS2, and MSH6, MSH2 (figures 1,2,3 and 4). The results showed that a significant proportion of cases (out of 64 cases) exhibited loss of expression of at least one of these proteins:

· MLH1: 24.2% (15 out of 62 cases analyzed).

· PMS2: 25% (15 out of 60 cases analyzed).

· MSH6: 14.5% (9 out of 62 cases analyzed).

· MSH2: 12.7% (8 out of 63 cases analyzed).

Statistically, there were no significant correlations between age and MLH1 (p-value 0.113), MSH2 (p-value 0.07), MSH6 (p-value 0.055), and PMS2 (p-value 0.065).

## Discussion

Endometrial cancer is the most common gynecological malignancy worldwide, and it is classified into two main types based on histology features: type I (estrogen-dependent, often well-differentiated) and type II (estrogen-independent, often poorly differentiated) [7,8].

The molecular classification of endometrial carcinoma has been refined in recent years, with TCGA research network identifying four distinct molecular subgroups based on genomic alterations [8]. TCGA is a landmark cancer research program that has had a major impact on our understanding of cancer genomics. It was a comprehensive, multi-year effort led by the National Institutes of Health to systematically analyze the molecular basis of major cancer types. It involved the collection and analysis of tumor samples from thousands of cancer patients across the United States. For endometrial cancer specifically, the TCGA study published a landmark paper in 2013 that classified endometrial carcinomas into four major molecular subtypes based on their genomic profiles. This classification has become a widely adopted framework for understanding the heterogeneity of endometrial cancer and has significantly advanced research and clinical management in this disease.

First group is the POLE-ultramutated: This subgroup is characterized by tumors with extremely high mutational burden, driven by mutations in the exonuclease domain of the POLE gene. These tumors tend to be high-grade, but have an overall favorable prognosis [9].

Second group is microsatellite instability-hypermutated (MSI): This subgroup encompasses tumors with MMR deficiency, leading to widespread microsatellite instability. As discussed earlier, these tumors often exhibit distinct histopathological features [10].

The third group is copy-number low (CN-low)/p53-wildtype: This subgroup is characterized by tumors with a relatively low number of CN alterations and intact p53 function. They are typically low-grade endometrioid carcinomas with a good prognosis. For example, patients with CN-low/p53-wildtype endometrial tumors often present with early-stage, well-differentiated endometrioid adenocarcinomas. These tumors tend to have a low mutational burden and lack the aggressive genomic features seen in the CN-high or p53-abnormal subtypes. As a result, patients in the CN-low/p53-wildtype group generally have excellent overall survival outcomes compared to the other molecular subgroups.

Last group is CN-high/p53-abnormal: This subgroup includes tumors with extensive CN alterations and p53 abnormalities, often corresponding to the more aggressive type II endometrial carcinomas, such as serous and clear cell subtypes [11].

This molecular classification has important clinical implications, as each subgroup is associated with distinct clinicopathological features, prognosis, and potential therapeutic vulnerabilities [12,13]. Incorporating these molecular markers into the routine evaluation of endometrial carcinomas can help refine risk stratification and guide personalized management strategies for patients. The molecular classification of endometrial carcinoma has been a topic of intense research, and MMR deficiency has emerged as a critical factor in understanding the heterogeneity of this disease [14]. MMR proteins play a crucial role in maintaining genomic stability by correcting DNA replication errors. Deficiencies in these proteins lead to an accumulation of mutations, known as the MSI phenotype, which is characteristic of a subset of endometrial carcinomas [14]. MMR deficiency, which results in MSI, is found in approximately 25-30% of endometrial cancers [14]. This MMR-deficient subtype is more commonly seen in type I endometrial cancers. In comparison, MSI is found in around 15-20% of colorectal cancers and 4-20% of gastric cancers, making endometrial cancer one of the cancer types with the highest prevalence of MMR deficiency and MSI.

Assessing the MMR protein panel is an important step in the molecular characterization of endometrial cancers. The MMR protein panel typically includes IHC staining for the four key MMR proteins: MLH1, MSH2, MSH6, and PMS2 [15].

IHC analysis of the MMR protein panel can be used to infer the underlying MMR deficiency status of the tumor. Loss of expression of one or more MMR proteins, as detected by the absence of nuclear staining, is indicative of MMR deficiency [16]. This MMR-deficient phenotype is often associated with the presence of MSI in the tumor, which can be confirmed by additional molecular testing [17].

Importantly, the pattern of MMR protein loss can provide clues about the specific underlying genetic mechanism responsible for the MMR deficiency. For example, isolated loss of MLH1 protein expression is frequently associated with epigenetic silencing of the MLH1 gene through promoter hypermethylation, whereas concurrent loss of MSH2 and MSH6 proteins is more commonly seen in cases of germline mutations in the MSH2 gene [18,19]. Understanding the specific MMR deficiency mechanism can have implications for genetic counseling and clinical management of patients with endometrial cancer.

Overall, the assessment of the MMR protein panel by IHC is a valuable tool in the comprehensive molecular profiling of endometrial tumors, with important diagnostic and prognostic implications.

Numerous studies have highlighted the distinct clinicopathological features associated with MMR-deficient endometrial carcinomas. These tumors are more likely to exhibit higher tumor grades, specific histological subtypes (such as endometrioid carcinoma), and increased lymphovascular space invasion [20]. Importantly, MMR deficiency has also been linked to poorer clinical outcomes, including higher rates of recurrence, metastasis, and worse overall survival [21].

Interestingly, MMR-deficient endometrial cancers tend to occur at a younger age compared to MMR-proficient tumors [22]. Several studies have reported a median age of diagnosis around 55-60 years for MMR-deficient endometrial cancers, which is about 5-10 years younger than the typical age of diagnosis for endometrial cancer overall [22]. This younger age of onset highlights the importance of considering MMR status when managing endometrial cancer patients.

The prevalence of MMR deficiency in endometrial cancer also appears to vary by ethnicity. Some studies have reported a higher frequency of MMR deficiency in endometrial tumors from Asian and Hispanic/Latina women compared to White or African American women [22]. For example, one study found MMR deficiency in 41% of endometrial cancers in Asian women, 35% in Hispanic/Latina women, 26% in White women, and 18% in African American women [6]. These potential ethnic disparities in MMR deficiency rates underscore the need to consider patient demographics when screening for this molecular feature.

The histopathological features of MMR-deficient endometrial carcinomas have been well-characterized in the literature. These tumors often exhibit distinct morphological characteristics that can aid in their identification [22].

MMR-deficient endometrial carcinomas are more commonly of the type I, estrogen-dependent subtype, and they tend to be well to moderately differentiated [22,23]. Histologically, these tumors are characterized by a solid, glandular, or papillary growth pattern, with the presence of tumor-infiltrating lymphocytes being a common feature [24,25]. The neoplastic cells may show clear, hobnail, or secretory cytoplasmic features, and they often exhibit high-grade nuclear atypia, including prominent nucleoli and frequent mitotic figures [25].

Interestingly, a subset of MMR-deficient endometrial carcinomas may display a "serrated" morphology, reminiscent of the serrated pathway of colorectal carcinogenesis [26,27]. These tumors are characterized by the presence of glands with a sawtooth-like appearance, as well as the presence of eosinophilic cell secretions, which can further aid in their histological recognition [26,27].

The distinct histopathological features of MMR-deficient endometrial carcinomas not only have diagnostic value but may also have prognostic implications, as these tumors have been associated with improved clinical outcomes compared to their MMR-proficient counterparts [27,28].

The identification of MMR deficiency in endometrial cancer is clinically relevant, as it can help guide treatment decisions and identify individuals at risk for Lynch syndrome, an inherited condition that predisposes to certain cancers [29]. Overall, the younger age of onset and potential ethnic differences in MMR deficiency rates in endometrial cancer emphasize the importance of a personalized approach to patient management.

The identification of MMR-deficient endometrial carcinomas has important therapeutic implications. These tumors exhibit increased neoantigen load and enhanced immune infiltration, rendering them potentially more responsive to immune checkpoint inhibitor therapies [29]. Studies have shown that MMR-deficient endometrial cancers have improved response rates and progression-free survival when treated with pembrolizumab, a PD-1 inhibitor, compared to MMR-proficient tumors [30].

Moreover, MMR status can also guide the selection of other targeted therapies. For example, MMR-deficient endometrial cancers may benefit from PARP inhibitor treatment due to the increased genomic instability associated with this molecular subtype [31]. Clinical trials are ongoing to evaluate the efficacy of combination strategies, such as immune checkpoint inhibitors plus PARP inhibitors, in this patient population [32].

The recognition of the prognostic significance of MMR status in endometrial cancer has led to the recommendation of routine MMR testing for all patients with this disease [32]. This assessment not only informs treatment decision-making but also facilitates the identification of individuals with Lynch syndrome, a hereditary condition that predisposes individuals to multiple cancer types, including endometrial cancer [32].

Early detection of Lynch syndrome is crucial, as it allows for targeted surveillance and preventive strategies for affected individuals and their families. This can significantly improve patient outcomes and reduce the burden of cancer [32]. Furthermore, the identification of MMR-deficient endometrial carcinomas may also guide the selection of targeted therapies, such as immune checkpoint inhibitors, which have demonstrated promising results in this molecular subtype of the disease [33].

In addition to its prognostic and therapeutic implications, the assessment of MMR status in endometrial carcinoma has important implications for genetic counseling and cancer screening. Identifying MMR deficiency can indicate the presence of Lynch syndrome, a hereditary condition that increases the risk of multiple cancer types, including endometrial cancer [33]. This information is crucial for the patient and their family members, as it allows for appropriate surveillance and prevention strategies.

In summary, the incorporation of MMR status evaluation into the routine diagnostic and management workflow for endometrial carcinoma has become increasingly important. This molecular characteristic not only provides valuable prognostic information but also guides the selection of targeted therapies and facilitates the identification of individuals with hereditary cancer syndromes, ultimately improving patient outcomes and reducing the burden of this disease.

Limitations

This study has several important limitations that should be considered when interpreting the findings. Firstly, it was a single-center study conducted at a major tertiary care hospital in Bahrain. While this facility serves a large proportion of the national population, the findings may not be fully representative of the entire country. A multi-center study encompassing data from various healthcare institutions would provide a more comprehensive overview of endometrial cancer epidemiology in Bahrain.

Additionally, not all endometrial cancer specimens underwent IHC evaluation for MMR protein expression. The MMR status was only available for a subset of the study cohort. This limited the ability to definitively determine the overall prevalence of MMR deficiency in the Bahraini endometrial cancer population. Routine implementation of universal MMR testing would allow for more accurate assessment of this important biomarker.

Furthermore, the retrospective nature of the study design relied on the availability and completeness of medical records, which may have been subject to missing or inaccurate data. Prospective data collection with standardized protocols would help overcome these limitations and provide more robust evidence.

Lastly, the study did not explore potential risk factors, such as obesity, diabetes, and reproductive history, which have been associated with endometrial cancer development. Inclusion of these variables in future research would enable a more comprehensive understanding of the underlying etiological factors driving the observed patterns of endometrial cancer in Bahrain.

Despite these limitations, the current study provides valuable insights into the epidemiological and clinicopathological characteristics of endometrial cancer in the Bahraini population. The findings contribute to the existing body of knowledge and underscore the need for larger-scale, multi-institutional investigations to further elucidate the burden and management of this important gynecological malignancy in the country.

## Conclusions

This study found that endometrial cancer is a significant health concern in Bahrain, with a relatively high prevalence compared to global averages. The data indicates that endometrial cancer in Bahrain tends to occur in younger age groups, is more commonly diagnosed at higher grades, and exhibits a predominance of the endometrioid histological subtype. Additionally, a notable proportion of cases had MMR deficiency, which is linked to increased risk and more aggressive disease. These findings align with broader global trends of rising endometrial cancer incidence, particularly among younger women, suggesting the need for enhanced screening, early detection, and tailored treatment approaches in Bahrain.

Further research is warranted to fully understand the underlying risk factors and molecular features driving the observed endometrial cancer patterns in Bahrain. 

## References

[1] Bokhman JV (1983). Two pathogenetic types of endometrial carcinoma. Gynecol Oncol.

[2] Felix AS, Weissfeld JL, Stone RA, Bowser R, Chivukula M, Edwards RP, Linkov F (2010). Factors associated with type I and type II endometrial cancer. Cancer Causes Control.

[3] Hoang LN, McConechy MK, Köbel M (2013). Histotype-genotype correlation in 36 high-grade endometrial carcinomas. Am J Surg Pathol.

[4] Niu S, Molberg K, Castrillon DH, Lucas E, Chen H (2024). Biomarkers in the diagnosis of endometrial precancers. Molecular characteristics, candidate immunohistochemical markers, and promising results of three-marker panel: current status and future directions. Cancers (Basel).

[5] Kandoth C, Schultz N, Cherniack AD (2013). Integrated genomic characterization of endometrial carcinoma. Nature.

[6] Bray F, Ferlay J, Soerjomataram I, Siegel RL, Torre LA, Jemal A (2018). Global cancer statistics 2018: GLOBOCAN estimates of incidence and mortality worldwide for 36 cancers in 185 countries. CA Cancer J Clin.

[7] Lortet-Tieulent J, Ferlay J, Bray F, Jemal A (2018). International patterns and trends in endometrial cancer incidence, 1978-2013. J Natl Cancer Inst.

[8] (2024). Cancer Stat Facts: Uterine Cancer. https://seer.cancer.gov/statfacts/html/corp.html.

[9] Henry CE, Phan K, Orsman EJ, Kenwright D, Thunders MC, Filoche SK (2021). Molecular profiling of endometrial cancer: an exploratory study in Aotearoa, New Zealand. Cancers (Basel).

[10] Elming PB, Wittenborn TR, Busk M (2021). Refinement of an established procedure and its application for identification of hypoxia in prostate cancer xenografts. Cancers (Basel).

[11] Li JY, Park HS, Huang GS, Young MR, Ratner E, Santin A, Damast S (2021). Prognostic impact of mismatch repair deficiency in high- and low-intermediate-risk, early-stage endometrial cancer following vaginal brachytherapy. Gynecol Oncol.

[12] Maddalo D, Manchado E, Concepcion CP (2014). In vivo engineering of oncogenic chromosomal rearrangements with the CRISPR/Cas9 system. Nature.

[13] Stelloo E, Bosse T, Nout RA (2015). Refining prognosis and identifying targetable pathways for high-risk endometrial cancer; a TransPORTEC initiative. Mod Pathol.

[14] Llosa NJ, Cruise M, Tam A (2015). The vigorous immune microenvironment of microsatellite instable colon cancer is balanced by multiple counter-inhibitory checkpoints. Cancer Discov.

[15] Latham A, Srinivasan P, Kemel Y (2019). Microsatellite instability is associated with the presence of Lynch syndrome pan-cancer. J Clin Oncol.

[16] Konstantinopoulos PA, Ceccaldi R, Shapiro GI, D'Andrea AD (2015). Homologous recombination deficiency: exploiting the fundamental vulnerability of ovarian cancer. Cancer Discov.

[17] Fader AN, Roque DM, Siegel E (2018). Randomized phase II trial of carboplatin-paclitaxel versus carboplatin-paclitaxel-trastuzumab in uterine serous carcinomas that overexpress human epidermal growth factor receptor 2/neu. J Clin Oncol.

[18] Hampel H, Frankel WL, Martin E (2005). Screening for the Lynch syndrome (hereditary nonpolyposis colorectal cancer). N Engl J Med.

[19] Zhao S, Chen L, Zang Y (2022). Endometrial cancer in Lynch syndrome. Int J Cancer.

[20] Shia J (2008). Immunohistochemistry versus microsatellite instability testing for screening colorectal cancer patients at risk for hereditary nonpolyposis colorectal cancer syndrome. Part I. The utility of immunohistochemistry. J Mol Diagn.

[21] Bartosch C, Manuel Lopes J, Oliva E (2011). Endometrial carcinomas: a review emphasizing overlapping and distinctive morphological and immunohistochemical features. Adv Anat Pathol.

[22] Gologan A, Sepulveda AR (2005). Microsatellite instability and DNA mismatch repair deficiency testing in hereditary and sporadic gastrointestinal cancers. Clin Lab Med.

[23] Simpkins SB, Bocker T, Swisher EM (1999). MLH1 promoter methylation and gene silencing is the primary cause of microsatellite instability in sporadic endometrial cancers. Hum Mol Genet.

[24] Ip PP, Cheung AN, Clement PB (2009). Uterine smooth muscle tumors of uncertain malignant potential (STUMP): a clinicopathologic analysis of 16 cases. Am J Surg Pathol.

[25] Garg K, Soslow RA (2014). Endometrial carcinoma in women aged 40 years and younger. Arch Pathol Lab Med.

[26] Shia J, Black D, Hummer AJ, Boyd J, Soslow RA (2008). Routinely assessed morphological features correlate with microsatellite instability status in endometrial cancer. Hum Pathol.

[27] Zighelboim I, Goodfellow PJ, Gao F, Gibb RK, Powell MA, Rader JS, Mutch DG (2007). Microsatellite instability and epigenetic inactivation of MLH1 and outcome of patients with endometrial carcinomas of the endometrioid type. J Clin Oncol.

[28] Bülbül G, Aktaş TÇ, Aysal Ağalar A, Aktaş S, Kurt S, Saatli B, Ulukuş EÇ (2024). Morphomolecular correlation and clinicopathologic analysis in endometrial carcinoma. Int J Gynecol Pathol.

[29] Yamamoto H, Imai K (2015). Microsatellite instability: an update. Arch Toxicol.

[30] Talhouk A, McConechy MK, Leung S (2015). A clinically applicable molecular-based classification for endometrial cancers. Br J Cancer.

[31] Matrai C, Motanagh S, Mirabelli S (2020). Molecular profiles of mixed endometrial carcinoma. Am J Surg Pathol.

[32] Talhouk A, McAlpine JN (2016). New classification of endometrial cancers: the development and potential applications of genomic-based classification in research and clinical care. Gynecol Oncol Res Pract.

[33] Murali R, Soslow RA, Weigelt B (2014). Classification of endometrial carcinoma: more than two types. Lancet Oncol.

